# Effect of rK39 testing in guiding treatment initiation and outcome in patients with visceral leishmaniasis in Ethiopia: A prospective cohort study

**DOI:** 10.1371/journal.pone.0253303

**Published:** 2021-06-14

**Authors:** Dawit Gebreegzabher Hagos, Yazezew Kebede, Mahmud Abdulkader, Etsay Nigus, Zekarias Gessesse Arefaine, Gebreab Nega, Henk D. F. Schallig, Dawit Wolday

**Affiliations:** 1 Mekelle University College of Health Sciences, Mekelle, Ethiopia; 2 Department of Medical Microbiology, Parasitology Unit, Amsterdam University Medical Center, Amsterdam, The Netherlands; National Centre For Cell Science, INDIA

## Abstract

**Background:**

The rapid diagnostic test (RDT) rK39 is currently being used for routine diagnosis of visceral leishmaniasis (VL) in East Africa. However, continuous monitoring of the performance of the assay, in particular its impact on the clinical decision in initiating anti-leishmanial treatment and outcomes remains needed as there are concerns about the diagnostic performance of this test.

**Methods:**

VL patients prospectively enrolled in a diagnostic trial and with rK39 RDT were included. We evaluated the effect of rK39 testing in guiding treatment initiation and outcome. On the basis of rK39 RDT test result as well as clinical case definition for VL and microscopy examination, the clinicians decide whether to initiate VL therapy or not. Poisson regression models were used to identify factors associated with a decision to initiate VL therapy. In addition, treatment outcomes of those who received VL therapy were compared to those who received non-VL treatment.

**Results:**

Of 324 VL suspects enrolled, 184 (56.8%) were rK39+ and 140 (43.2%) were rK39‒. In addition, microscopy exam was done on tissue aspirates from a sub-population (140 individuals), which is 43.2% of the suspected cases, comprising of 117 (63.6%) rK39+ and only 23 (16.4%) rK39‒ cases. Of those with microscopy examination, only 87 (62.1%) were found positive. Among 184 (56.8%) patients without microscopy, 67 (36.4%) were rK39+, of whom 83 (65.9%) were positive by microscopy, 21 (16.7%) were negative by microscopy and 22 (17.5%) had no microscopy results. On the other hand, of those who did not receive VL treatment 58/189 (30.7%) were rK39+ and 131 (69.3%) were rK39‒. Of the rK39+ cases who did not receive VL therapy, only 1 (1.7%) patient was microscopy positive, 12 (20.7%) were negative and 45 (77.6%) patients had no microscopy result. Of the rK39‒ cases (n = 131) who did not receive VL treatment, 16 were microscopy negative and 115 without microscopy exams. Whereas positive rK39 result [adjusted Relative Risk (aRR) 0.69; 95% CI: 0.49–0.96, p = 0.029] and positive microscopy results (aRR 0.03; 95% CI: 0.00–0.24, p = 0.001) were independently associated with VL treatment, having confirmed diagnosis other than VL (aRR 1.64; 95% CI: 1.09–2.46, p = 0.018) was independently associated with initiation of non-VL therapy. The proportion of rK39+ patients who received non-VL treatment with no improvement outcome was significantly higher when compared to those who received VL treatment (24.1%, 95% CI: 14.62–37.16 vs. 11.9%, 95%CI: 7.26–18.93; p<0.0001).

**Conclusion:**

The study shows that a significant proportion of patients with rK39+ results were undertreated. Failure to do microscopy was associated with lack of improved clinical outcome. Including an additional simple point-of-care assay in the diagnostic work-up is urgently needed to correctly identify VL cases and to prevent morbidity and mortality associated with the disease.

## Introduction

Leishmaniasis remains one of the World’s most neglected infectious diseases (NIDs). The World Health Organisation (WHO) estimates that 350 million people in 98 countries are at risk of contracting leishmaniasis and approximately 1.5 million new cases occur yearly [1]. Visceral leishmaniasis (VL), or Kala Azar, is endemic in many countries in Eastern Africa, including Ethiopia, where up to 4,000 cases are reported annually, and an estimated 5 million people live at risk of being infected [2]. Ethiopia is one of the seven countries in which more than 90% of global VL cases occur and one of the ten countries with the highest estimated case counts, which together account for 70 to 75% of global estimated VL incidence [1]. Ethiopia has been recently listed by the WHO among the fourteen countries in the world with the highest burden of VL [3]. VL, caused by *L*. *donovani* is an increasing public health problem in Ethiopia, with endemic areas that are continually spreading [4–7]. HIV-*Leishmania* co-infection in Ethiopia is one of the highest (40%) in the world [8–11], though recent data show a trend towards reduction around 21–25% [12].

Untreated VL can cause severe morbidity and ultimately leading to death in almost all cases. Accurate and prompt diagnosis of VL is thus essential to initiate treatment immediately. In several VL endemic areas, including Ethiopia, current standard diagnosis is based on clinical findings such as fever, organomegaly and weight loss, confirmed by direct parasite detection in clinical specimens, such as bone marrow, splenic or lymph node aspirates. Though microscopy is widely used and has been used successfully in remote areas, its sensitivity declines with decreasing numbers of parasites, which is commonly noted in VL patients [13, 14]. Splenic aspirate is the most sensitive (>90%), but dangerous for the patient, and the diagnostic sensitivity in other aspirates is lower than that of the spleen [15]. Bone marrow aspiration/biopsy is a common method of obtaining a sample for microscopy examination, but its sensitivity ranges between 60–85% [15]. In addition, bone marrow aspiration/biopsy is painful and can be complicated by infection. Lymph node aspirate has low sensitivity around 54%-65% [16–18]. Overall, these aspiration/biopsy procedures are invasive, technically not feasible and with complications in the most remote parts where the disease is endemic. The rK39 rapid diagnostic test (RDT) which has been extensively evaluated and used for the diagnosis of VL in Asia and East Africa [19–27]. Furthermore, rK39 has become a standard diagnostic approach for the diagnosis of VL in East Africa, including Ethiopia, as part of an algorithm that includes direct-agglutination test (DAT) and microscopy examination [28].

Though rK39 assay is being used currently for routine diagnosis of VL in East Africa, continuous monitoring of the performance of the assay, in particular its impact on the clinical decision on initiating anti-leishmanial treatment is needed. Diro E et al. [29] described that the national VL diagnostic algorithm in Gondar, Northern Ethiopia, is not strictly adhered to and a significant proportion of the rK39 tested cases (~61%) did not undergo microscopy exam. In addition, subsequent diagnostic efforts made and non-VL treatments provided were not known for most of these patients in whom rK39 were positive or those with negative results and without microscopy confirmation. Consequently, this study aims to undertake a detailed evaluation of the effect of rK39 testing in guiding treatment initiation and outcome in patients with VL in an endemic area in Northern Ethiopia.

## Methods

### Study site and participants

This study was undertaken between June 2019 and October 2020 at Ayder Generalized Referral Hospital, affiliated to Mekelle University College of Health Sciences, Mekelle City, in Tigray Regional State, Northern Ethiopia. The study is part of the EvaLAMP project, a prospective cohort study evaluating the utility of loop-mediated isothermal amplification assay (LAMP) for VL diagnosis (Clinicaltrials.gov: NCT04003532).

Patients from endemic area and presenting with clinical case definition criteria suggestive of VL (including fever > 2 weeks, weight loss, splenomegaly [28]) were enrolled prospectively in the study. Patients were excluded if they received treatment with any anti-leishmanial drugs within the previous 3 months, not capable of understanding or complying with the study protocol, or refusal to consent and participate into the study. Suspected VL cases were tested using rK39 RDT, and microscopic examination of Giemsa stained splenic or bone marrow aspirates for the presence of *L*. *donovani* amastigotes. In addition, patients were tested for HIV-1 co-infection using HIV RDTs.

Anti-VL therapy was based on the National Guideline for Diagnosis, Treatment and Prevention of Leishmaniasis in Ethiopia [28], and included treatment with a combination of sodium stibogluconate (SSG) 20 mg/kg body weight/day) plus paromomycin (15 mg/kg body weight/day), both given intramuscularly for 17 consecutive days if HIV uninfected. In the absence of or stock-out of paromomycin, SSG was given as monotherapy (20 mg/kg body weight/day) for 30 days. Liposomal amphotericin B (AmBisome) was provided as first-line therapy for HIV-1 co-infected patients or pediatric age group given at 5mg/kg body weight/day over a period of 6 consecutive days. Amphotericin B deoxycholate was given at 1 mg/kg body weight as 15 alternate-day infusions over 30 days in the event AmBisome was not available.

### Sample size

We assumed that the proportion of VL cases among patients without microscopy examination is higher among patients not started on anti-VL treatment compared to those started on non-VL treatment. With an expected 50% prevalence of VL among suspects, 80% power to detect a 10% difference in the proportion of VL cases between the two groups, a desired binomial error margin of ± 5% and an α level of 0.05, we estimated a minimum sample size of 260 VL suspects in order to identify 130 confirmed VL and 130 non-VL cases. Taking into account an anticipated response rate of 90%, we aimed to recruit a total sample size of 290 VL suspects (145 VL cases and 145 non-VL cases) as defined by clinical case definition [28].

### Serology

For all the subjects, rK39 RDT was performed from blood samples according to manufacturer’s instruction (IT LEISH, Bio-Rad Labs, The Netherlands). Screening for HIV-1 was done using STAT-PACK (Chembio Diagnostic Systems Inc., New York, USA) RDT based on National Guidelines [30]. Those positive for HIV-1 with screening test were then confirmed by a second test ABON (Abon Biopharma, Hangzhou, China), and in the event of discrepant result, a tie-breaker with SD Bioline (SD Standard Diagnostics Inc., Seoul, South Korea) RDT was done.

### Definitions

Clinical case definition for VL was defined as having fever, weight loss, splenomegaly or hepatomegaly, and pancytopenia [28]. Pancytopenia was defines as having WBC count <3500/μL, or hemoglobin level of <11 gm/dL, or platelet count of <120 000/μL [31]. Improved clinical outcome is defined a combination of the following: resolution of fever, weight gain, regression of organomegaly and improvement in laboratory parameters, in particular pancytopenia.

### Statistical analysis

Baseline characteristics for continuous variables were expressed as the median with interquartile range (IQR), and for categorical variables as proportions. Chi-square test was used to compare categorical variables and Wilcoxon rank-sum test for not normally distributed variables. Factors associated with probability of initiating VL therapy were associated using Poisson regression analysis. In univariate analysis, the probability [Relative Risk (RR)] of not receiving anti VL therapy was analyzed with respect to several socio-demographic, clinical and laboratory markers. Then, multivariate analysis of probability [adjusted Relative Risk (aRR)] of not receiving VL therapy was calculated by including all variables that were significant in univariable analysis. A test was considered significant if p value was < 0.05. Statistical analysis was carried out using STATA statistical software version 14.0 (StataCorp, Texas, USA).

### Ethics statement

The study was reviewed and approved by the Health Research Ethics Review Committee of Mekelle University College of Health Sciences (ERC#: 1102/2017), and the National Research Ethics Review Committee, Ministry of Science and Higher Education. Written informed consent was obtained from all study participants or legal guardians.

## Results

### Characteristics of the study population

A total of 324 VL suspects were enrolled (Table 1). Median age of study participants was 24 years (IQR: 18–35) and the majority were male (90.7%). Most of the patients presented with fever, weight loss, organomegaly, pancytopenia, and the majority (88.6%) fulfilled the clinical case definition for VL [28]. Three-fifth of all the VL suspects (60.2%) had co-morbid conditions or primary disease other than VL, such as HIV-1, tuberculosis, malaria, other infectious diseases as well as non-communicable diseases (NCDs) (Table 1). A total of 39 (28.9%) VL suspects who received VL therapy had co-morbid conditions. In addition, 156 (82.5%) of those who received non-VL therapy had a diagnosis of confirmed non-VL disease and in the remaining 17.5%, diagnosis was not ascertained. Whereas HIV was more frequent among patients who received VL therapy, conditions such as tuberculosis, malaria, other bacterial infections and NCDs were much more common among those who received non-VL therapy.

**Table 1:** Characteristics of the study population stratified by visceral leishmaniasis (VL) treatment status.

Characteristic	All VL suspects (n = 324)	VL treatment (n = 135)	No VL treatment (n = 189)	*p* value
**Age [median years (IQR)]**	24 (18–35)	23 (17–30)	27 (18–39)	0.0125
**Sex**				
Male	294 (90.7)	125 (92.6)	169 (89.4)	
Female	30 (9.3)	10 (7.4)	20 (10.6)	0.331
**VL symptoms**				
Fever	313 (96.6)	132 (97.8)	181 (95.6)	0.325
Weight-loss	300 (92.6)	132 (97.8)	168 (88.9)	0.003
Fatigue	275 (84.9)	130 (96.3)	145 (76.7)	<0.0001
Abdominal swelling	220 (67.9)	126 (93.3)	94 (49.4)	<0.0001
**Clinical signs**				
Temp. [median (IQR) °C]	37.6 (36.9–38.2)	38.0 (37.3–38.8)	37.2 (36.8–37.8)	<0.00001
Temperature ≥ 37.3°C	184 (56.8)	100 (74.1)	84 (44.4)	<0.0001
Splenomegaly	246(75.9)	127 (94.1)	119 (62.9)	<0.0001
Hepatomegaly	101 (31.2)	56 (41.5)	45 (23.8)	0.001
**Laboratory markers**				
WBC total [median (IQR)/ μL]	2200 (1500–3900)	1700 (1200–2200)	3225 (2000–4900)	<0.00001
Hemoglobin [median (IQR) mg/dL]	8.1 (6.3–10.7)	7.5 (6.3–8.7)	8.7 (6.3–11.9)	0.0002
Hematocrit [median (IQR) %]	24.4 (18.6–31.2)	22.7 (18.4–26.4)	27.7 (19.1–35.1)	0.0002
Platelet count [median (IQR)/μL]	183000 (101000–382000)	115000 (70000–231000)	211000 (144000–460000)	<0.00001
SGOT [median (IQR) U/L]	54 (32–111)	82 (49–152)	44 (29–73)	<0.00001
SGPT [median (IQR) U/L]	33 (20–54)	44 (30–73)	26 (17–41)	<0.00001
Alkaline phosphate [median (IQR) U/L]	123 (74–203)	147 (105–234)	117 (65–186)	0.0634
Bilirubin-direct [median (IQR) mg/dL]	0.47 (0.27–0.95)	0.52 (0.32–1.40)	0.44 (0.25–0.72)	0.031
BUN [median (IQR) mg/dL]	23 (17–30)	22 (15–27)	23 (18–33)	0.1203
Creatinine [median (IQR) mg/dL]	0.63 (0.50–0.79)	0.59 (0.43–0.76)	0.67 (0.56–0.80)	0.011
rK39+ RDT	184 (56.8)	126 (93.3)	58 (30.7)	<0.0001
Microscopy examination:				
Not done	184 (56.8)	24 (17.8)	160 (84.7)	<0.0001
Done	140 (43.2)	111 (82.2)	29 (15.3)	<0.0001
Parasite positive	87 (62.1)	86 (77.5)	1 (3.5)	<0.0001
Clinical case definition for VL	287 (88.6)	128 (94.8)	159 (84.1)	0.003
Follow-up duration [median days (IQR)]	9 (6–18)	17 (8–23)	8 (5–12)	<0.00001
**Comorbidity or other disease**				
Any disease	195 (60.2)	39 (28.9)	156 (82.5)	<0.0001
HIV	38 (11.7)	24 (17.8)	14 (7.4)	0.004
Tuberculosis	11 (3.4)	1 (0.7)	10 (5.3)	0.026
Malaria	37 (11.4)	3 (2.2)	34 (18.0)	<0.0001
Bacterial co-infections*	40 (12.4)	4 (3.0)	36 (19.1)	<0.0001
NCDs**	46 (14.2)	2 (1.5)	44 (23.3)	<0.0001
**Death outcome**	13 (4.0)	9 (6.7)	4 (2.1)	<0.0001

HIV: human immunodeficiency virus; NCDs: non-communicable diseases; VL: visceral leishmaniasis

*Co-infections (pneumonia, meningitis, urinary tract infections, sepsis.

**NCDs (cardiovascular diseases, chronic kidney disease, chronic liver disease, and malignancy).

**Fig 1:**
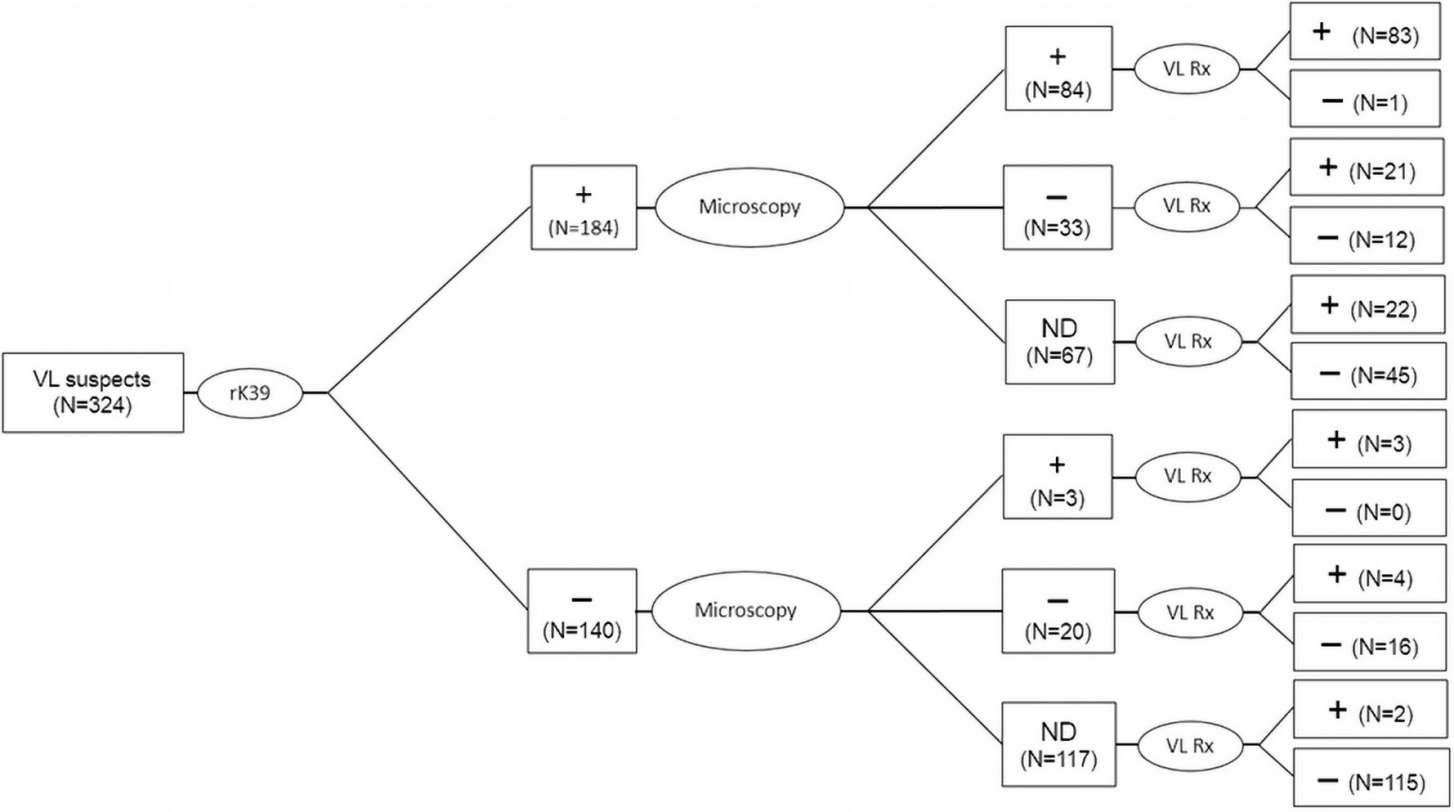
Flow chart of the clinical algorithm for management of visceral leishmaniasis. Abbreviations: VL = visceral leishmaniasis; ND = not done; N = number of cases; Rx = VL treatment.

Among the 324 VL suspects who were enrolled, 184 (56.8%) were rK39+ and 140 (43.2%) were rK39‒ (Fig 1). Of these, Giemsa’s staining microscopy for amastigote identification was done from bone marrow or spleen aspirates in only 140 (43.2%), who included 117 (63.6%) rK39+ and only 23 (16.4%) rK39‒ cases. Of the 140 VL suspects on whom microscopy was done, 87 (62.1%) were positive for *L*. *donovani* amastogotes; of which 84 were also positive by rK39, giving a sensitivity of 96.6% (95% CI: 90.25–99.28) and 3 (2.1%) were rK39‒. On the other hand 53 (37.9%) were microscopy negative of which 33 were rK39+ and 20 were rK39‒, giving a specificity of 37.7% (95% CI: 24.79–52.11) for rK39 RDT. Of the 184 (56.8%) VL suspects without microscopy test, 67 (36.4%) were rK39+ and the remaining 117 (63.6%) were rK39‒.

### Factors associated with initiation of anti-VL treatment

VL treatment was provided for a total of 135 (41.7%) VL suspects and the remaining 189 (58.3%) received non-VL treatment (Table 1). Though there was no difference in gender, those who were initiated with VL therapy were significantly younger [23 (IQR: 17–30) vs. 27 (IQR: 18–39) years, p = 0.0125]. Fever was common in both; but significant proportion of VL suspects who presented with weight loss, fatigue, abdominal swelling, high temperature, splenomegaly, hepatomegaly, pancytopenia and abnormal LFTs were initiated with VL treatment when compared to those who received non-VL management. On the other hand abnormal RFTs were more frequent among those who received non VL therapy. There were more patients that fulfilled the clinical case definition for VL among those who were initiated VL therapy when compared to those who received non-VL treatment [94.8% (95% CI: 89.43–97.53) vs. 84.1% (95% CI: 78.14–88.71), p = 0.003]. In addition, duration of follow-up was significantly longer for those who received VL therapy compared to those who received non-VL treatment. Of the total 135 VL suspects who received VL therapy, 126 (93.3%) were rK39+, of whom 83 (65.9%) were positive by microscopy, 21 (16.7%) were negative by microscopy and 22 (17.5%) had no microscopy results (Fig 1). In addition, VL was given to only 9 patients who were rK39‒. Of these, 3 (33.3%) were positive and 4 (44.4%) were negative by microscopy, while in 2 (22.2%) microscopy was not done. Among those who were prescribed VL therapy, 63 (46.7%) received ambisome, 42 (31.1%) received SSG plus paromomycin, 28 (20.7%) received SSG, and only 2 (1.5%) received Amphotericin-B. On the other hand, of the total 189 patients who received non-VL treatment 58 (30.7%) were rK39+ and 131 (69.3%) were rK39‒. Of the rK39+ cases, only 1 (1.7%) patient was positive by microscopy, 12 (20.7%) were negative and 45 (77.6%) patients had no microscopy exam results available. Of the rK39‒ cases, a total of 131 patients, 16 were microscopy negative and 115 without microscopy exams.

Factors associated with initiation of VL treatment is summarized in Table 2. Whereas positive rK39 result (aRR 0.69; 95% CI: 0.49–0.96, p = 0.029) and positive microscopy results for parasite (aRR 0.02; 95% CI: 0.00–0.16, p = 0.001) were independently associated with VL treatment, having confirmed diagnosis other than VL (aRR 1.64; 95% CI: 1.09–2.46, p = 0.018) was independently associated with initiation of non-VL therapy.

**Table 2:** Factors associated with initiation of non-VL therapy.

Characteristic	Crude Relative Risk	P value	Adjusted Relative Risk	P value
Male gender	0.86 (0.54–1.37)	0.531		
Age group (years)				
<25	Ref.		Ref.	--
25–45	1.12 (0.82–1.52)	0.455	1.07 (0.76–1.50)	0.714
>45	1.61 (1.02–2.54)	0.043	1.08 (0.65–1.78)	0.778
Fever	0.80 (0.39–1.61)	0.526		
Weight-loss	0.60 (0.39–0.94)	0.025	0.90 (0.55–1.46)	0658
Fatigue	0.59 (0.42–0.82)	0.002	1.02 (0.69–1.52)	0.920
Abdominal swelling	0.47 (0.35–0.62)	<0.0001	0.79 (0.54–1.15)	0.221
Temperature (≥37.3 °C)	0.60 (0.45–0.79)	<0.0001	0.95 (0.69–1.32)	0.758
Splenomegaly	0.54 (0.40–0.72)	<0.0001	1.05 (0.70–1.57)	0.829
Hepatomegaly	0.69 (0.49–0.96)	0.030	0.88 (0.59–1.30)	0.513
WBC (per μL)				
3500–10000	Ref.	--	Ref.	--
<3500	0.53 (0.39–0.72)	<0.0001	0.87 (0.62–1.22)	0.408
>10000	0.92 (0.42–1.99)	0.829	0.99 (0.44–2.23)	0.991
Hemoglobin (<11 mg/dL)	0.55 (0.40–0.75)	<0.0001	1.04 (0.72–1.50)	0.838
Platelet count (<120000/μL)	0.43 (0.29–0.64)	<0.0001	0.93 (0.61–1.40)	0.720
SGOT (<40 U/L)	0.71 (0.49–1.02)	0.065		
SGPT (<56 U/L)	0.66 (0.41–1.10)	0.106		
Alkaline phosphate (<140 U/L)	0.75 (0.38–1.50)	0.419		
Bilirubin-direct (<0.3 mg/dL)	0.72 (0.42–1.25)	0.244		
BUN (<20 mg/dL)	1.12 (0.74–1.70)	0.597		
Creatinine (<1.21 mg/dL)	1.29 (0.74–2.25)	0.370		
rK39+ serology	0.34 (0.25–0.46)	<0.0001	**0.69 (0.49–0.96)**	**0.029**
Parasite microscopy examination:				
Negative	Ref.	--	Ref.	--
Positive	0.02 (0.00–0.16)	<0.0001	**0.03 (0.00–0.24)**	**0.001**
Not done	1.65 (1.10–2.46)	<0.015	1.19 (0.75–1.87)	0.465
Clinical case definition for VL	0.68 (0.46–1.01)	0.056		
Other disease confirmed				
Any disease	3.13 (2.15–4.55)	<0.0001	**1.64 (1.09–2.46)**	**0.018**
NCDs	1.83 (1.31–2.57)	<0.0001	1.12 (0.78–1.62)	0.536
HIV	0.60 (0.35–1.04)	0.068		
Tuberculosis	1.59 (0.84–3.01)	0.154		
Malaria	1.70 (1.17–2.47)	0.005	1.34 (0.90–1.99)	0.152
Infection	1.67 (1.16–2.40)	0.006	1.23 (0.83–1.82)	0.304

HIV: human immunodeficiency virus; NCDs: non-communicable diseases; VL: visceral leishmaniasis

### Treatment outcomes

Results of treatment outcomes is summarized in Table 3. VL therapy was given to a total of 135 (41.7%) patients and the remaining 189 (58.3%) received non-VL treatment. Of those put on VL treatment, 119 (88.1%) completed therapy while 16 did not (2 transferred, 9 died, and 5 discharged against medical advice). Of those who completed standard therapy, follow-up assessment demonstrated that 112 (83.0%) patients improved after VL therapy, and the remaining 11.1% and 5.9% had unfavorable or unknown outcome, respectively. Among those who received VL therapy with improved clinical outcome, there were 104 (82.5%) rK39+ cases and 74 (86.1%) microscopy-confirmed VL cases. Of the total 185 cases who did not receive VL therapy, 107 (56.6%) has favorable clinical outcome, but 43 (22.8%) and 39 (20.6%) had unfavorable or unknown outcome, respectively.

**Table 3:** Outcomes stratified by VL laboratory diagnosis result status and VL treatment status.

Outcomes	VL treatment [N = 135 (41.7%)]			Non VL treatment [N = 189 (58.3%)]		
	Improved	Not improved	Unknown	Improved	Not improved	Unknown
All cases	112 (83.0%)	15 (11.1%)	8 (5.9%)	107 (56.6%)	43 (22.8%)	39 (20.6%)
rK39+	104 (82.5%)	15 (11.9%)	7 (5.6%)	30 (51.7%)	14 (24.1%)	14 (24.1%)
Microscopy +	74 (86.1%)	8 (9.3%)	4 (4.7%)	-	-	1 (100%)
rK39+/microscopy+	71 (85.5%)	8 (9.6%)	4 (4.8%)	-	-	1 (100%)
rK39+/microscopy‒	14 (66.7%)	5 (23.8%)	2 (9.5%)	9 (75.0%)	2 (16.7%)	1 (8.3%)
rK39+/microscopy not done	19 (86.4%)	2 (9.1%)	1 (4.6%)	21 (46.7%)	12 (26.7%)	12 (26.7%)
rK39‒/microscopy+	3 (100%)	-	-	-	-	-
rK39‒/microscopy‒	3 (75.0%)	-	1 (25.0%)	9 (56.3%)	3 (18.8%)	4 (25.0%)
rK39‒/microscopy not done	2 (100%)	-	-	68 (59.1%)	26 (22.6%)	21 (18.3%)

Overall, the proportion of unfavorable clinical outcome among those who were provided with non VL therapy was significantly higher when compared with those who received VL treatment (22.8%, 95% CI: 17.28–29.33 vs. 11.1%, 95%CI: 6.77–17.72; p<0.0001). Furthermore, the proportion of rK39+ patients who received non-VL treatment with no improvement outcome was significantly higher when compared to those who received VL treatment (24.1%, 95% CI: 14.62–37.16 vs. 11.9%, 95%CI: 7.26–18.93; p<0.0001). Likewise, the proportion of rK39+ VL suspects on whom parasite examination was not done and who received non-VL treatment with no improvement in outcome was significantly higher when compared to those similar group of cases who received VL treatment (26.7%, 95% CI: 15.46–41.96 vs. 9.1%, 95%CI: 2.02–32.65; p<0.0001). There were 13 deaths, of which 9 (69.2%) received VL therapy. In these nine cases, causes of death were drug toxicity in two cases and cardiac arrest, bleeding, liver failure, multi-organ failure, and respiratory arrest. The cause of death in two cases was not known. There was no statistically significant difference in mortality between those who received VL therapy when compared with those who received non-VL therapy.

## Discussion

Accurate, timely and rapid diagnosis is needed to reduce morbidity and mortality associated with VL. The traditional diagnosis of VL in resource-constrained settings is based on microscopy and rK39 RDT. Nonetheless, both methods are faced with significant inherent problems making them suboptimal for the diagnosis of VL. Previously undertaken meta-analysis demonstrated that the sensitivity of rK39 test was significantly lower in East Africa [85.3%; 95% confidence interval (CI): 74.5 to 93.2] than in the Indian subcontinent (97.0%; 95% CI: 90.0 to 99.5), despite no significant difference in specificity. The specificity in East Africa and the Indian subcontinent was 91.1% (95% CI: 80.3 to 97.3) and 90.2% (95% CI: 76.1 to 97.7), respectively [20]. Furthermore, the positive predictive value (PPV) and negative-predictive value (NPV) of rK39 in East Africa is 93% and 81%, respectively [20]. However, in a clinical setting in Ethiopia, the PPV of rK39 was found to be much lower, around 69.6% [21]. The differences in the results of the various evaluations undertaken so far are related to smaller sample size, differences in methods used, including nature of the specimen, as well as the differences employed as reference standards and control groups and different diagnostic kits used [19–26]. In addition, differences in test performances might be related to parasite heterogeneity and differential anti-leishmanial IgG responses [32–34]. On the other hand, antibodies against rK39 may persist in the circulation in asymptomatic infections [35], or previously infected patients for many months or years after the parasites have been cleared [36, 37], making detection of active infection inaccurate.

Although traditionally specificity is assessed by testing specimens from patients with other diseases or from healthy controls for *Leishmania* infection, it may be more relevant to describe specificity in terms of the absence of active VL disease (i.e. microscopy and/or PCR negative). Ideally, if microscopy of tissue aspirate is considered as reference standard, then a significant proportion of patients (7–44%) with rK39 test results and presumed as false-positive would indeed be true-positive given that microscopy’s sensitivity ranges between 54–93% [15–18]. Our findings support the above notion, as we found low specificity in our cases (20/53 = 37.7%), because of the 33/53 (62.3%) microscopy negative cases were found to be rK39+ (or false positives). The fact that the majority [31 (93.9%) of the 33 cases] had no past history of VL indicates that the rK39 positivity was not due to persistent antibodies against rK39. It also concurs with our previous report showing a specificity of 34% for rK39 [26], and also suggestions made earlier that the inclusion of reference controls from suspected VL from a consecutively and prospectively enrolled subjects shows significantly lower estimates for specificity when compared to controls enrolled from healthy individuals [31]. Furthermore, additional testing of microscopy negative results using molecular methods have been shown to improve the specificity of rK39 in VL cases [38, 39].

National guidelines for VL treatment in Ethiopia dictate to treat all patients who fulfill clinical case definition of VL and rK39+ [28]. Nonetheless, in our cohort, 38/189 (20.1%) of those fulfilling the diagnostic criteria for VL [13 (34.2%) of which were also rK39+] did not receive VL therapy and treatment outcome with non-VL treatment was not favorable. It suggests that these group probably would have benefitted from VL therapy. Furthermore, when rK39 RDT results become negative, then VL suspects should undergo DAT that is not available or should have microcopy exam done. In our cohort, of the total 140 VL suspects whose rK39 RDT was negative, only 23 had microscopy examination (3 were positive and 20 were negative). However, in the majority of the patients (83.6%) microscopy was not done. Of the later, 26/115 (22.6%) patients did not exhibit clinical improvement following non-VL treatment, suggesting that there might be a possibility of underestimation in VL diagnosis among these cases. If rK39 positivity is considered in combination with the clinical case definition, and given a PPV of 93% for rK39 RDT for identifying true VL cases in Eastern Africa [20], then a total of 171 of the 184 rK39+ would receive VL treatment. In addition, given the NPV of rK39 at 81% for East African VL [20], 27 of the 140 rk39‒ cases should have received VL therapy. Overall, a total of 198 VL suspects should have been treated. Nonetheless, in our cohort, only 135 (68.2%) were treated for VL and the remaining 31.8% did not receive VL therapy despite the majority being rK39+ and fulfilling clinical case definition. In addition, only 140 (43.2%) had parasite exam done (63.6% for rK39+ and for only 16.4% for rK39‒ cases). This is somewhat similar to the previous report from Ethiopia [11]. The fact that a significant proportion of rK39+ patients in our cohort who were put under non-VL treatment option exhibited poor treatment outcome significantly when compared to those who received VL treatment suggests also that the patients would probably have benefitted from VL therapy based on the rK39 results.

The strengths of the study include the prospective nature of the study and the assessment of treatment outcomes of those who received non-VL therapy. However, small sample size and lack of parasitology exam for all VL suspects are some of the limitations of the current study.

In conclusion, a large proportion of rK39+ patients who fulfil the diagnostic criteria for active VL remained untreated and those with rK39‒ results did not undergo further microscopy exam resulting in exaggerated or under treatment for VL of the cohort. Further studies are necessary to estimate the exact magnitude of missed VL cases among those fulfilling clinical case definition by adding additional tests, including molecular assays [38, 40, 41]. In our set-up we are currently evaluating the utility of the LAMP assay for improving the diagnosis of VL. Rapid and accurate diagnosis of VL would help reduce morbidity and mortality significantly.
